# Multi-modal medical image classification using deep residual network and genetic algorithm

**DOI:** 10.1371/journal.pone.0287786

**Published:** 2023-06-29

**Authors:** Muhammad Haris Abid, Rehan Ashraf, Toqeer Mahmood, C. M. Nadeem Faisal

**Affiliations:** Department of Computer Science, National Textile University, Faisalabad, Pakistan; Mirpur University of Science and Technology, PAKISTAN

## Abstract

Artificial intelligence (AI) development across the health sector has recently been the most crucial. Early medical information, identification, diagnosis, classification, then analysis, along with viable remedies, are always beneficial developments. Precise and consistent image classification has critical in diagnosing and tactical decisions for healthcare. The core issue with image classification has become the semantic gap. Conventional machine learning algorithms for classification rely mainly on low-level but rather high-level characteristics, employ some handmade features to close the gap, but force intense feature extraction as well as classification approaches. Deep learning is a powerful tool with considerable advances in recent years, with deep convolution neural networks (CNNs) succeeding in image classification. The main goal is to bridge the semantic gap and enhance the classification performance of multi-modal medical images based on the deep learning-based model ResNet50. The data set included 28378 multi-modal medical images to train and validate the model. Overall accuracy, precision, recall, and F1-score evaluation parameters have been calculated. The proposed model classifies medical images more accurately than other state-of-the-art methods. The intended research experiment attained an accuracy level of 98.61%. The suggested study directly benefits the health service.

## 1. Introduction

In recent years, multiple medical CBIR techniques have been presented. The majority of developed CBIR retrieval mechanisms employ a single imaging modality. The retrieval algorithms can give the chance to choose the image class before similarity comparison which is one technique to retrieve the required medical images across large image libraries. A CBIR system might benefit greatly from good image categorization since it would eliminate the desire to search through irrelevant images, reducing the number of images that the system would have to look [1].

Convolutional neural networks (CNN) established approaches not only improve classification accuracy, although they are also considered good general descriptors of features. CNN extracts features in a hierarchical manner, with lower layers encoding lower characteristics such as edges, forms, texture, and so on, and higher levels encoding semantic level aspects associated with an image. Because the kernels within these networks have been learned rather than constructed, no preliminary parameterize nor human involvement is required [1].

Non-learning approaches perform better under some situations; nonetheless, the disparity across higher levels semantics with low-level depictions of features in diverse pictures leads in reduced image retrieval efficiency. Existing techniques have also used multiple learning-based strategies to close the gaps in semantics that increase picture retrieval effectiveness. Despite techniques using learning effectively bridge the gap within higher levels semantics alongside low-level visualizations of features across various images, they are dependent upon several kinds of various attributes and lack the ability to perform adequately on all types of images over each feature descriptor. Furthermore, learning-based CBIR approaches are computationally challenging than non-learning CBIR techniques [2].

Convolutional neural networks (CNNs) have already made significant advancements in the computer vision field [3]. There have already been introduced multiple neural network architectures, such as VGGNet, GoogLeNet, ResNet, DenseNet, as well as more recently NASNet [4]. ResNet itself and its variants have drawn the most attention within these deep networks. ResNet has shown exceptional results across both medium and high computer vision applications. This shortcut connection technique, which enables the training about a deeper structure where gradients may directly flow across construction blocks but the gradient vanishing dilemma can be somewhat avoided, is largely responsible for ResNets’ exceptional success. Its shortcut connection process, on the other hand, forces every block to concentrate on learning its residual output while somehow ignoring the internal block connectivity and making it therefore that some reusable knowledge generated in earlier blocks is often neglected in subsequent blocks [5].

The current CBIR studies are focused on developing new techniques to better describe visual material that are more relevant to the users. In recent trends for medical image retrieval investigations, the images are described using a set of semantic terms. This set of semantically specified image attributes could be applied to recognize a broad variety of images which increases the user’s attention to visual aspects [6, 7]. The advantages of semantic terms for diagnostics decision-making would be that they could enable radiologists to search image databases across instances with similar high-level and enhanced quality [8].

The radiologist’s keywords for image observations are the key factor that is appropriate for the implementation of CBIR [9, 10]. To bridge the semantic gap across images and their associated meaning, adding semantics to image description can therefore be a novel technique [11]. This combination with text attribute searching, which depends on the contents of the image, with limited visual features, which are generated directly first from the representation of images, has been discovered to enhance medical semantic search findings [12].

In this study, we stated the issue of semantic gap elimination in image retrieval. To resolve this challenge, semantic language for radiological image contents has been proposed. Feature searches and visual qualities complement each other. Complementary notions and implementation to image databases results in a more will useful result for all users. These visual characteristics of an image transmit a relatively low level of characterization, making it impossible to accurately express using keywords alone.

The major contributions of this proposed work are:

➢ To bridge the semantic gap across user requests with system responses
➢ To implement a genetic algorithm for optimal multi-classification
➢ To apply optimal model training for improving the multi-classification of multimodal medical images

## 2. Related works

In the last few years, digital image processing and the combination of machine learning has shown good results in various applied domains of computer vision [13–15]. The recent focus of research for image classification-based models is the use of deep learning architectures and frameworks [16–18].

In the work of [19], the primary goal of the modality classification included separate various forms of medical images, such as X-ray, CT, electrocardiography, and PET, as well as generic graphs, from other medical sources for illness diagnosis. Radiologists require an effective categorization system for retrieving associated clinical cases to make accurate illness diagnoses [20]. Similarly [21] used the Faster-RCNN technology in together with SVM-based classifier to provide a unique strategy for the autonomous classification about melanoma lesions. If the challenge is one of classification, it may be preferable to utilize a deep neural network [17].

The development of medical image modality by [22] categorization system was beneficial in narrowing the retrieval query space within a specific modality. For the creation of modality categorization systems, two techniques were frequently utilized i) hand-crafted (traditional) but also ii) Deep neural networks. Medical images, especially as opposed to general images, contain a variety of characteristics such as postural difficulties, texture, and aesthetic elements. The classification of medical image modalities had been mostly based on form, color, and textured features [23]. Similarly [13] discussed positive guiding importance towards multi-modal medical imaging evaluation.

Deep Learning techniques were [24] suggested the best alternative for classifying medical images. Algorithms are used to automatically extract the primary elements from medical images in order to execute the classification procedure. The primary concept is to create feature maps using Conv layers. During the convolution operations, various filter masks having varied orientations are employed to build feature maps. Such maps are then processed using pooling procedures as a feature minimization method. The goal is to employ the most realistic characteristics for image classification and avoid any manually extracted features throughout the process of classification. In work of [16] increased the adaptability about deep inpainting structures to training sets alongside diverse variety, while improving inpainting effectiveness as judged through qualitative as well as quantitative measures for an extensive variety about deep models.

Researchers have presented [25] modality classification algorithms to improve performance on baseline methods published by Image CLEF. It was noted that the effectiveness of previously proposed techniques employing hand-crafted attributes varies but achieves adequate accuracy overall [26]. It was because classification performance is heavily reliant on expert judgment when obtaining acceptable data for modality categorization. This was challenging to determine the amount and kind of retrieved attributes from modality images for efficient categorization [27, 28]. Those techniques were limited by their large computing needs as well as the constraint of conditionality. Mostly as a result, it is essential to design an efficient modalities categorization strategy that enhances performance while requiring less human interaction.

Regarding image retrieval under a multi-class instance, a CBIR technique utilizing a hybrid characteristics descriptor using the genetic algorithm alongside SVM classifier was presented by [2]. The suggested method’s performance was evaluated using four benchmark datasets, alongside its comparison to 25 alternative CBIR approaches. Experimental findings show that their technique surpasses prevailing state-of-the-art retrieval algorithms.

Several CNN models with binary along with multi-class categorization of COVID-19 instances were studied by [29]. These models were tested on various CT alongside X-ray datasets using Transfer learning ideas for deep-tuning while fine-tuning settings. Transfer learning frameworks involving LeNet-5, VGG16, AlexNet, along with Inception nave v1 being deep-tuning frameworks and DenseNet121, DenseNet201, DenseNet169, ResNet50, VGG16, ResNet152, and VGG19 being fine-tuning frameworks have been thoroughly compared. Simulation tests were carried out on a total of 12,032 images from chest CT with X-ray collections (COVID-19 = 2,466, pneumonias = 4,273, and normal = 5,293). Every model was evaluated using a variety of categorization assessment measures. Considering the investigated X-ray with CT images, ResNet152 and DenseNet201 performed better compared to various Transfer learning frameworks. Similarly investigated [14] improved deep learning based LeNetsþþ on softmax, centred integrating i-center loss function, using a variety of standard image recognition stages.

In the work of [30] presented a method for predicting patient survival based on reliability and effectiveness. Furthermore, researchers wanted to show how important it was to use classification then FS algorithms for achieving the greatest outcomes in the quickest period of time, since this is a critical aspect in an individual’s survival. Following doing trials and analyzing the findings with regard to of mistake rate and precision, it was revealed the classification algorithms delivers superior results when not combined alongside the FSFA. Therefore, rather than employing FSFAs, an approach based on classification proved more accurate and efficient.

The techniques’ scope was centred [31] on illness categorization, early screening, and organ localization, including benign and cancerous detection. Classification, and segmentation, including detection, are common CAD operations. Image classification treats each image only as a separate entity that must be distinguished from other images. Image separation was based upon pixel points, which divide the image over numerous distinct parts with distinct attributes, including image classification with the specific border of the existent objective [32]. Image detection seems to be the retrieval of a particular sub-image from a recognized image, whereas classification involves the retrieval of many items in an image [33].

The fundamental requirement for success mostly in classification explained by the [34] challenge was to identify highly discriminative characteristics about specific classes. This was very simple for categories having excellent internal consistency similarity, however, it may be challenging for domains having low inter-class correlation [35]. For example, mammography classification accuracy was generally poor, while discriminating characteristics for breast cancers are hard to capture in the context of overlapping, diverse fibro glandular structures. Considering the significant inter-class resemblance, the concept that fine-grained visual classification (FGVC), which tries to discover tiny distinctions among visually similar items, may be suitable for learning distinguishing characteristics [36].

As an outcome, techniques developed and assessed by [37] on such datasets could not be easily transferable for medical datasets when only a subset of images demonstrate significant inter-class similarities instead of all of them. Other methods for improving characteristic discrimination power incorporate the use of concentration modules, local and global features, specialized knowledge, and everything else [38]. If just a subset of the such training phase is labelled, the algorithm achieves the feedback connection from the labelled data but is enhanced through learning semantics plus fine-grained characteristics from the unsupervised learning [39]. As a result, the model optimizer was split into two stages: self-supervised pre-training but also supervised fine-tuning. The model was first improved using unidentified images to successfully learn excellent features which are indicative of such image semantics.

Although there were several approaches to constructing feature temples, a generally accepted rule seems to be that robust [40], moderate semantics must be combined alongside high-dimensional maps. Furthermore, when there were a high number of medical images that have structural, textural, but also semantic similarities with the targeted dataset, pre-training producers and/or classification techniques may help with computational efficiency and enhanced efficiency [41]. Similarly an effective transfer learning approach using the AlexNet framework provided [42] to properly classify and identify melanoma.

Following Table 1 is discussing and clarifying current studies, their limitation and helps us to bridge our research gap.

**Table 1:** Systematic literature review.

Publication Name & Ref	Contribution	Dataset and Techniques	Limitations and Future Work
Multimodal medical image segmentation using multi-scale context-aware network [43]	86.8% state-of the-art performance on three benchmark datasets of different modalities captures the rich context information with dense skip connection and assigns distinct weights to different channels	Multi-scale context-aware network (CA-Net) for multimodal medical image segmentation	Improve the feature expression ability of the network
Multimodal Medical Image Fusion Based on Pixel Significance Using Anisotropic Diffusion and Cross Bilateral Filter [44]	72% outperforms other algorithms in terms of objective evaluation metrics Edge preserving processing of the original images where it combines linear low pass filter with nonlinear techniques	Multimodal medical image fusion technique based on anisotropic diffusion and cross bilateral filter	Multimodal image fusion by proposing various algorithms
Efficient color image retrieval method using deep stacked sparse auto encoder [45]	Content-based image retrieval system for natural color images using a deep stacked sparse auto encoder	Corel-1K, Corel-10K DSSA model latent features	Efficacy of the latent representation learned
A deep neural network model for content- based medical image retrieval with multi-view classification [46]	Accuracy 92.35% Body part orientation view classification labels, intending to reduce the variance that occurs in different types of scans	A deep neural network-based approach for view classification and content-based image retrieval is proposed and its application for efficient medical image retrieval is demonstrated	Techniques to handle the class imbalance in the dataset with optimization techniques
Cross-Modality Sub-Image Retrieval using Contrastive Multimodal Image Representations [47]	Content-based image retrieval system (CBIR) for reverse (sub-)image search to retrieve microscopy images in one modality given a corresponding image captured by a different modality	Combine deep learning to generate representations that embed both modalities in a common space, with classic, fast, and robust feature extractors (SIFT, SURF) to create a bag-of-words model for efficient and reliable retrieval	Observe the importance of equivariance and invariance properties of the learned representations and feature extractors in the CBIR pipeline.
Content-based medical image retrieval using topic and location model [48]	Outperforms existing medical image retrieval systems in terms of Precision and Mean Average Precision. The proposed method achieved better Mean Average Precision (86.74%) compared	Automated medical image retrieval system using Topic and Location Mode	Retrieval of medical images based on the location and size of the anomaly
Content-based Image Retrieval and the Semantic Gap in the Deep Learning Era [49]	Semantic image retrieval Perform inferior to much less sophisticated and more generic methods	Aggregated convolutional features and opposed to traditional local features	Semantic image retrieval methods are often hardly comparable regarding the task definition and the evaluation data
Convolutional neural network-based dictionary learning to create hash codes for content-based image retrieval [50]	Visual complexity disappears	ResNet-50 architecture is trained with modified COREL dataset images	High retrieval time
MDCBIR-MF: Multimedia data for content-based image retrieval by using multiple features [9]	Qualitative methodology again for CBIR. Color values inside the HSV color space were utilized to extract color features, while texture characteristics were extracted using DWT and Gabor wavelets.	To improve the feature representation, the color, and edge omnidirectional descriptors were generated and incorporated, with widths of 1 250. As bigger the input vectors dimension, the much more precise the retrieval findings, but once longer it lasts to search and compare. The suggested system was evaluated on several datasets Corel and obtained good median accuracy.	High computational time owing to large feature vector dimensions.
An encrypted image retrieval method based on harris corner optimization and LSH in cloud computing [51]	Suggested a CBIR approach for retrieving encrypted images from the cloud that is predicated on an upgraded and the SURF detector with the descriptor.	These authors utilized the Local Sensitive Hash algorithm to generate accessible directories for extracted features to minimize retrieval time and improve	The performance is not great with large image datasets.

## 3. Methodology of proposed work

To assist our research, we used openly accessible medical datasets. This dataset contained five types of medical images (i.e. endoscopy, CT, chest, hand x-ray, and lungs CT). A maximum of 28378 good-quality jpg image formats was utilized within datasets. Images are then resized into 512 X 512 pixels. The model’s pre-processing procedure was used for the pre-processing purpose. Only as a consequence of our model’s testing using medical images, did researchers focus on establishing their database. Through this heterogeneous dataset, we picked images at irregular intervals from each class. During our research, we have used a dataset of 28378 images across 5 distinct classes. Crucial issues during this data included significant intra-class variance and great inter-class similarities caused by using multiple classes with various imaging technologies. We used 80% of the images during training and 20% throughout the testing. Because of the obtained dataset’s complex dimensions and structure, each image from each class was modified to 512 × 512 again and translated into a consistent jpg file. We used supervised learning to apply a class label.

**Fig 1:**
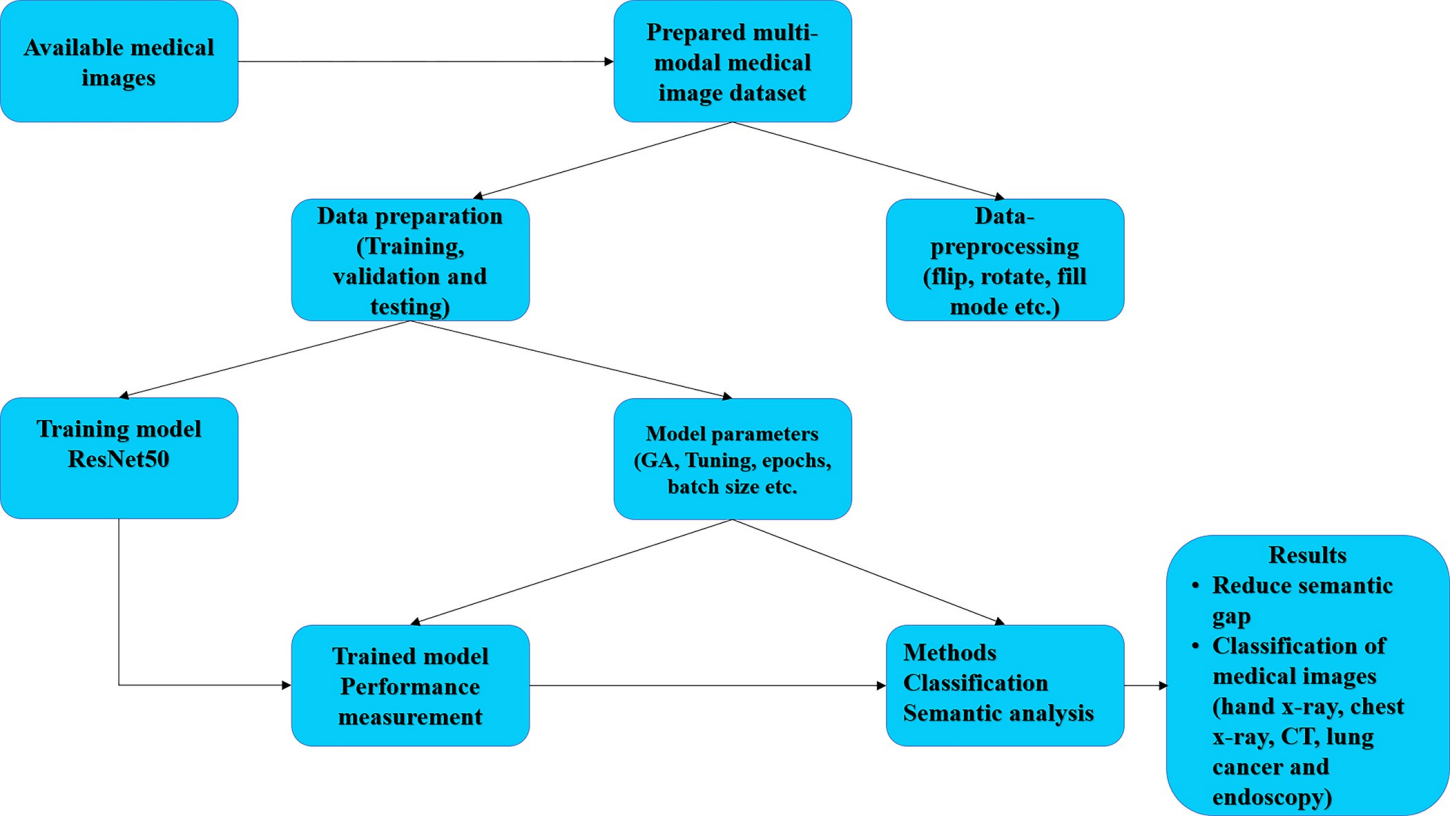
Proposed research flow diagram.

A possible perspective of multi-medical image classification and assessment is displayed in Fig 1. Images were initially gathered and sorted into classes. Image processing procedures include image shearing, transformations, image flipping, and scaling. These images were again input into the suggested method for model training at the next stage. That recently trained model has been used. Finally, multi-modal medical image identification but also classification had been achieved.

### 3.1 Images category

**Fig 2:**
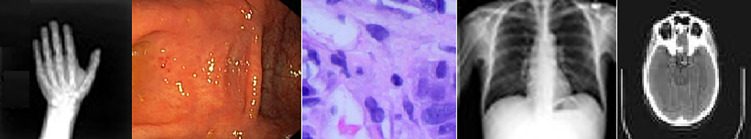
Sample medical images.

In this research, we used several medical images of multi-modal image classes which are shown in Fig 2. Generally, there are several steps of Machine Learning techniques toward medical image identification and classification employing Convolutional Neural Networks. These steps include dataset collection, dataset pre-processing, image segmentation, extraction of features, and classification. Each image was pre-processed and classified using the Kaggle platform. The significant percentage of datasets enhances the effectiveness of learning models and reduces over-fitting. Acquiring a dataset that can be used as input to such a training phase is a time-consuming but difficult task. As just a result, image enhancement expands the overall training data set offered for deep learning algorithms. Image flipping, resizing, rotation, color transformations, color enhancement, and noise reduction, are all deep learning-based intensification methodologies [52]. Automated extraction of features offers a high identification speed and precision. Feature extraction during segmentation converts the images towards a vector containing fixed features. These system-adopted characteristics include color, texture, but also shape. While extracting texture characteristics from some kind of color image, using a grey-scale cross matrix is preferable.

### 3.2 Genetic algorithm

We applied a Genetic algorithm for optimization. Genetic algorithms, which depend on bio-inspired operators including mutation, crossover, but also selection, are often employed to develop strong solutions for optimization and searching issues. The reason to use a genetic algorithm is that some greyscale medical images such as chest X-rays and CT need to be enhanced for better identification. Better identification will lead us toward optimized classification. By changing pixel values, the developed optimization algorithm will be reproduced dataset images. The implementation steps of the genetic algorithm included 1) reading of images 2) preparation of fitness function 3) implementation of mutation 4) implementation of statistics and results.

### 3.3 Transfer learning

The optimization with the training of the model seems to be a difficult yet time-consuming process. The training requires a strong graphics processing unit (GPU) along with thousands of training samples. Transfer learning, which is used in deep learning, meanwhile, eliminates all of these concerns. This transfer-learned per-trained Deep Learning Approach (CNN) is optimized for one activity and transfers information to different patterns [53]. This multi-modal images dataset model has 512 X 512 in size. We required modification in the residual network (ResNet). Its final layer even before softmax across all ResNet50 configurations is indeed a 7 X 7 average-pooling structure. Whenever a pooling size is reduced, a relatively small image may fit through into the network.

### 3.4 Convolutional neural network

**Fig 3:**
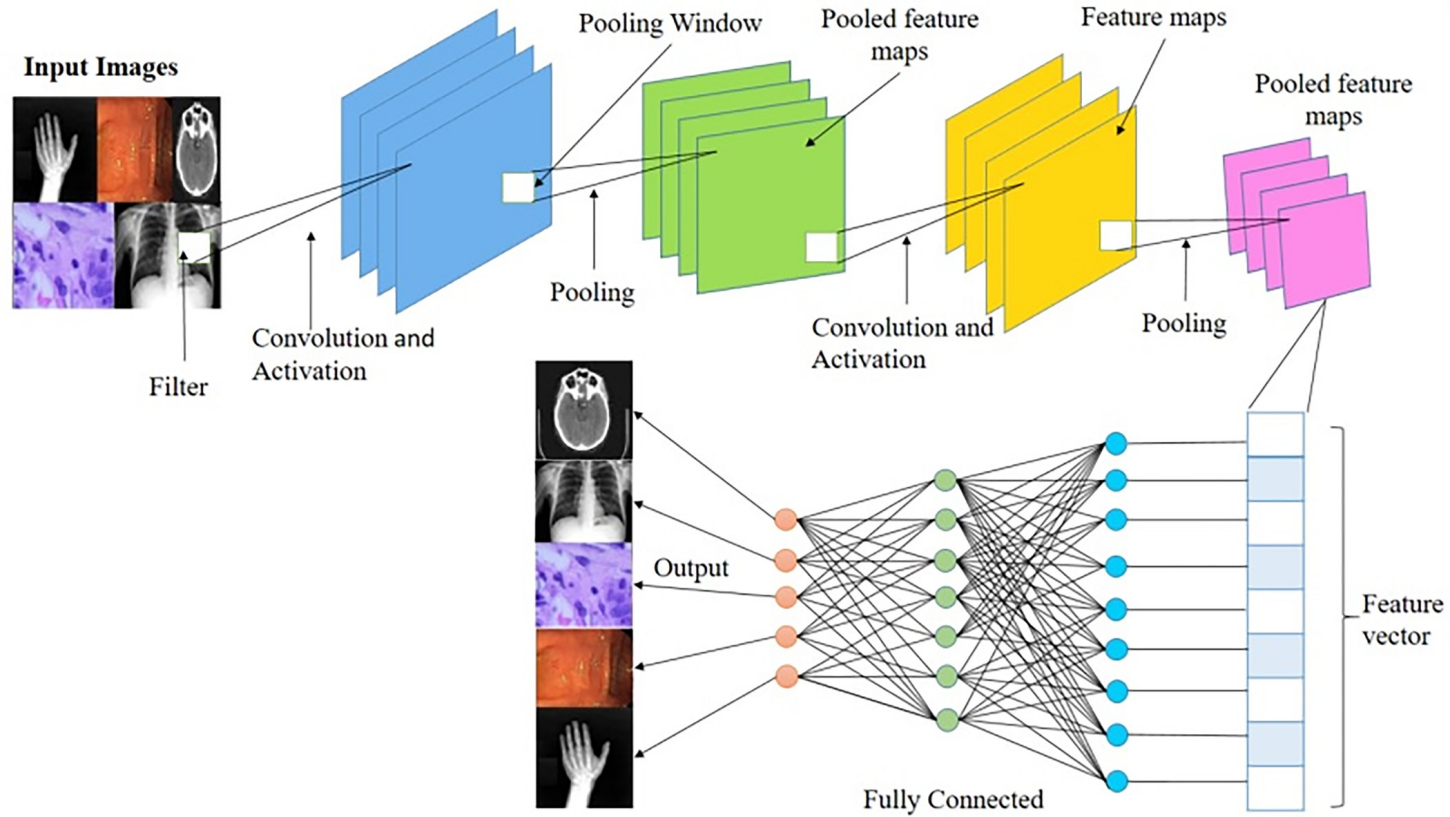
Proposed CNN architecture.

The structure of any Convolutional Neural Network (CNN) is made up of convolutional layers, pooling layers, and fully-connected layers, including dense layers as shown in Fig 3. The descriptions of the layers are presented below.

### 3.5 Convolutional layer

The primary function of convolutional layers included extracting distinctive features using images. The need for convolutional layers regularly aids throughout the extraction of input information [54]. The following Formula 1 is used to estimate the features extraction (FE_i_) across various layers through CNN.


$${FE}_{i}=\omega ({FE}_{i-1}{Wg}_{i}+{OFS}_{i})$$ (1)

Where, *FE_i_*—Feature map, *Wg_i_*–Weight, *OFS_i_* is offset and *ω*–Rectified Linear Unit (RELU).

### 3.6 Pooling layers

These pooling layers have become an important part of a Convolutional Neural Network (CNN). They reduce the dimensionality of convolved elements while also reducing the computer resources required for computer vision. Pooling may be divided into two categories maximum pooling plus average pooling. Usually, the highest values of images are returned by max pooling, but the mean values of such image sections are returned by average pooling.

### 3.7 Drop-out layers

Such dropout layers enhance the performance of a training phase. It offers regularization and inhibits over-fitting by lowering the correlation among neurons. Most activation functions employ the dropout procedure, however, it is enhanced by factor [55].

### 3.8 Flatten layers

It reduces its spatial dimensions about the mapping pooled characteristics while keeping its channel dimensions intact. This flattened layer includes dimensions before being converted into such a vector. This vectored input to completely linked layers is sometimes referred to here as a dense layer but rather fully connected layers.

### 3.9 Fully-connected layers

Along with their unique function, retrieved image categorization features require fully linked layers. This softmax function forecasts image properties collected from previous stages. Softmax is an activation function mostly in output layers that performs classification. During knowledge involvement, the neural network layer implements another multiplayer perceptron structure as either a classifier. Variability is induced in the entire vectors through the rectified linear unit (RELU) activated in the system. The depth of the ConvNet architectural design is its most important component. By establishing extra design parameters and continually increasing network depth by adding more convolutional layers, which is possible by employing extremely tiny convolution filters throughout all levels. Mostly as the outcome, have created significantly more precise ConvNet structures which not only achieve state-of-the-art precision on resolved input data classification as well as localization activities, while also being applicable towards other image processing datasets, within which they perform excellently even when used throughout relatively simple flow-lines.

Throughout the training, our ConvNets were provided this fixed-size 512 × 512 image. In only one pre-processing we have subtracted each pixel from the average value calculated mostly from the training dataset. To transport the image throughout a stacking of convolution operation, we use filters with an extremely tiny receptive field. In several of the setups, we also applied convolution filtering, which represents a linear modification of the inputs. This convolution stride was set to one pixel, and indeed the spatial padding of its convolutional layer inputs is set between one pixel for three convolution operations that maintain spatial resolution during convolutional. Spatial pooled is performed by 5 max-pooling levels that follow the portion of such convolutional layers.

### 3.10 ResNet50

ResNet50 pre-trained architectures using Convolutional Neural Networks are applied to increase performance but also classify images. This model adequately transfers information across pre-trained ResNet50 networks toward image quality identification and analysis. This CNN model has maintained fresh images learned to produce a model with identification and classification [56]. Using big kernel-sized filtering and convolutional layers besides a kernel filter size, our ResNet50 model improved. This size of the supplied image is set at 512 × 512. Images were pre-processed and then sent through another convolutional layer. This filter size was estimated with the linear treatment of the network interface (1 x 1). The stride value is taken at one, and the maximum pooling size was two by two. This filter size was specified for the sequential transformation of the channels. The fully—connected layer will use the same structure in the following phases, having 2048 channels within every layer. These Softmax activation structures are the outermost layer, succeeded by such RELU activation mechanisms in Table 2.

**Table 2:** Proposed Resnet50’s architecture.

Layer (type)	Output Shape	Param #	Connected to
input_1 (InputLayer)	[(None, 512, 512, 3)	0	Conv1
conv1_pad (ZeroPadding2D)	(None, 518, 518, 3)	0	input_1[0][0]
conv1_conv (Conv2D)	(None, 256, 256, 64)	9472	conv1_pad[0][0]
conv1_bn (BatchNormalization)	(None, 256, 256, 64)	256	conv1_conv[0][0]
conv1_relu (Activation)	(None, 256, 256, 64)	0	conv1_bn[0][0]
pool1_pad (ZeroPadding2D)	(None, 258, 258, 64)	0	conv1_relu[0][0]
pool1_pool (MaxPooling2D)	(None, 128, 128, 64)	0	pool1_pad[0][0]
conv2_block1_1_conv (Conv2D)	(None, 128, 128, 64)	4160	pool1_pool[0][0]
conv2_block1_1_bn (BatchNormali	(None, 128, 128, 64)	256	conv2_block1_1_conv[0][0]
conv2_block1_1_relu (Activation	(None, 128, 128, 64)	0	conv2_block1_1_bn[0][0]
conv2_block1_2_conv (Conv2D)	(None, 128, 128, 64)	36928	conv2_block1_1_relu[0][0]
………			
Total params: 26,733,446			
Trainable params: 3,145,734			
Non-trainable params: 23,587,712			
Number of FLOPs: 47175424/s			

## 4. Experiment, results, and discussion

The model has been fine-tuned to maximize accuracy with minimizing expected loss. On Kaggle, an extensive experimental analysis took place. Python programming packages have been uploaded since installed for scientific purposes. All experiments in our study were conducted under a computer including the following specifications: A CoreTM i7 CPU, 12 GB RAM, and a graphics card. This type of graphics card offers parallel computation throughout these training and testing periods. Upon that Windows 10 platform, Python (Keras plus Tensor Flow) was utilized to implement this whole training but also validation CNN methods. The data set has been structured as a directory containing two sub-directories, classes as well as tests. This classes directory is applied to training while the tests folder has been applied to testing. This class’s directory comprises five sub-directories containing various medical images (i.e. Endoscopy, CT, Chest, Hand X-ray, and Lung CT). Images categories were not allocated to the folders’ names. The purpose to achieve this is to effectively train set to bridge the semantic gap. The Directory structure can be explained by following Eqs 2 & 3.


$$f(I)=I_{MD}$$ (2)

$$I_{MD}=I_{MD}+\sigma$$ (3)

Where MD is a medical image collection and image denotes an image including a name but a path. Earlier than the training technique began, every single image within the dataset has been scaled into 512 × 512 x 3 during the pre-processing step. Eq 4 represents the scaling formula.


$$A_{I+1}=A_{I}+S_{X}$$

$$B_{I+1}=B_{I}+S_{Y}$$ (4)

The model has been loaded with adjusted weights after being fine-tuned based on dataset parameters. Every feature vector took into account the ultimate pooling layer’s conclusion. This pooling function involves applying a two-dimensional filtration to each channel from the feature map but then summarizing the features which lie within the filter’s covering zone. These are the dimensions that the output obtained because a pooling layer was used instead of a feature map well with dimensions provided in Eq 5.


(fmhi−fil+1)str*(fmwi−fil+1)stride*fmch (5)

Where *fmhi* is feature map height, *fmwi* is feature map width and *fmch* is the number of feature map channels. Similarly, *fi* is the size of the filter and *stride* is the length of the stride.

Given this decreasing gradient barrier, sigmoid and hyperbolic tangent activation has been utilized in multi-layer networks. Its rectified linear activation overcomes that vanishing gradients problem, allowing models to train faster while performing better. Utilizing rectified linear activation is the typical activation for developing multi-layer perceptron and convolutional neural networks. ReLU has been used here for activation functions in neural networks. ReLU is represented in Eq 6.


ReLU(Img)=max(0,Img) (6)

Whereas if the source becomes negative, then the result of ReLU equals 0; if the source becomes positive, then the result is *Img*.

Adam is one stochastic gradient optimizer. This common solution ’adam’ works well on moderately large datasets in respect of both management time plus validation scores. To pick activation or solver, a selected group has been made, i.e. returns a collection at random out of such an array. This random approach takes into account access to a variety of critical functions, including the capacity to generate random options.

In the next step genetic algorithm has been implemented for image reconstruction. The reason to use a genetic algorithm is that some greyscale medical images such as chest X-rays and CT need to be enhanced for better identification. Better identification leads us toward optimized classification. By changing pixel values, the developed optimization algorithm reproduced dataset images. The pixel levels varied within 0–255, 0–1 scale based upon that chromosomal description. This pixel-computed value influences other factors such as the range through which probabilities are chosen during mutation or the set of values utilized in the current population.

The code constructs one fitness function which will be used to calculate the overall fitness value with each solution within a population. Each function needs to be a maximizing function that receives two parameters, one indicating a solution while the second expressing its index. This gives back a value that represents the optimal solution. This fitness value can be calculated by adding the absolute differences in gene levels between the initial and replicated chromosomes. Since this genetic algorithm could work using 1D chromosomes, this function has been run before the actual fitness function should represent the image as such a vector. The fitness functions are represented in Eq 7.


FitnessFunction=1|x+y+z−t| (7)

Consider the following three factors: x, y, as well as z. The goal is to discover the optimum collection of parameters for x, y, but also z such that whose total value equals t. We must keep the total of x+y+z from departing from t, namely |x + y + z—t| must be zero. Only as result, the fitness value may be thought of as the inversion of |x + y + z—t|.

It is critical to employ random mutation but also set its mutation by replacement parameter to True. These bases for selecting towards the range low, range high, random mutation mini val, but also random mutation maxi val factors should be obtained based mostly on the range available pixel values. Whereas if image pixels are between 0 and 255, leave the range low and random mutation mini val at 0, but increase the range high with random mutation maxi val to 255. Mutation can be explained by Eq 8.


μ=mN (8)

Where N denotes the mean quantity of cells each cultured.

**Fig 4:**
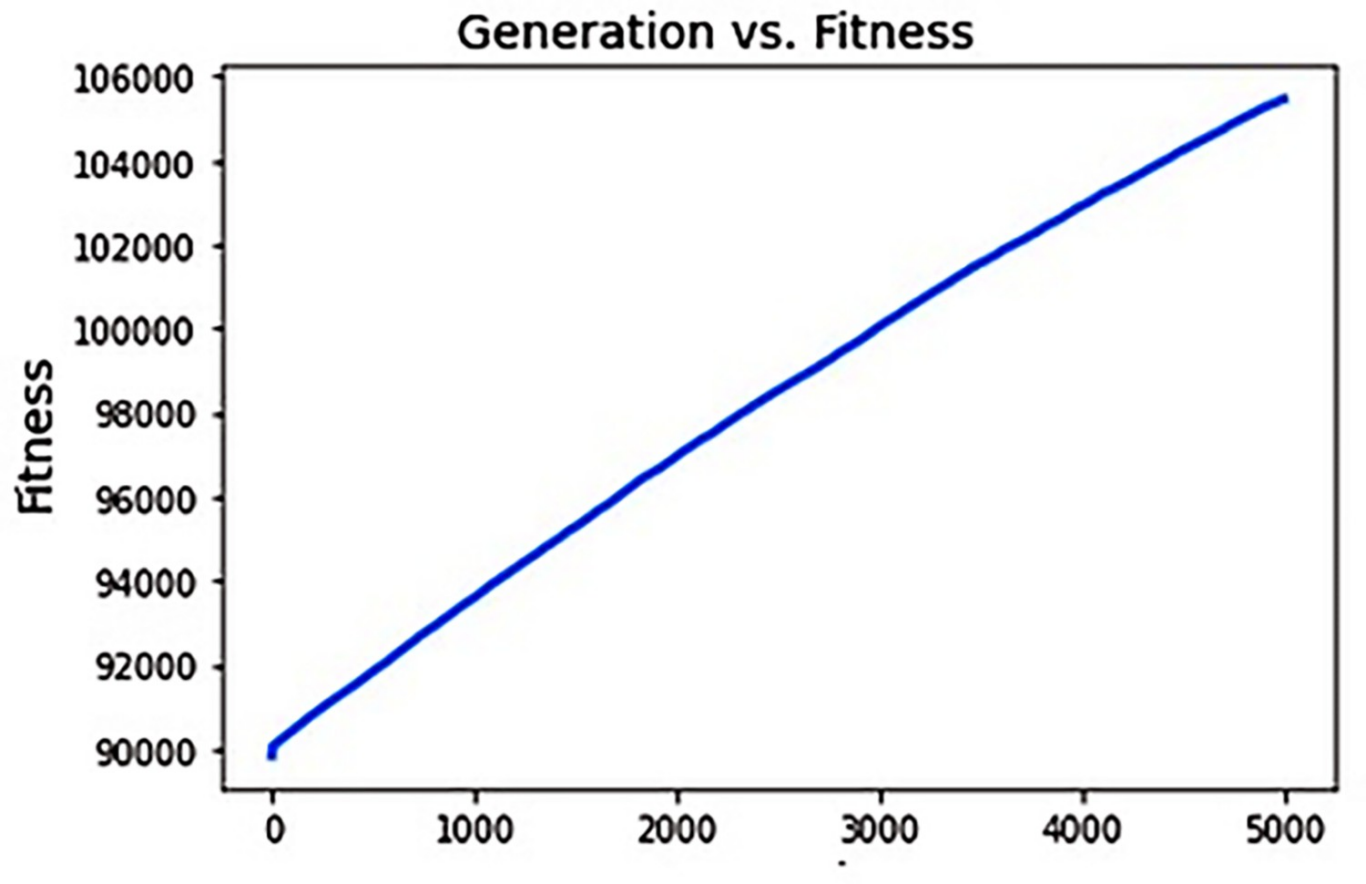
Fitness function.

Following the completion of the run procedure, actual fitness values among all generations may be observed in Fig 4.

**Fig 5:**
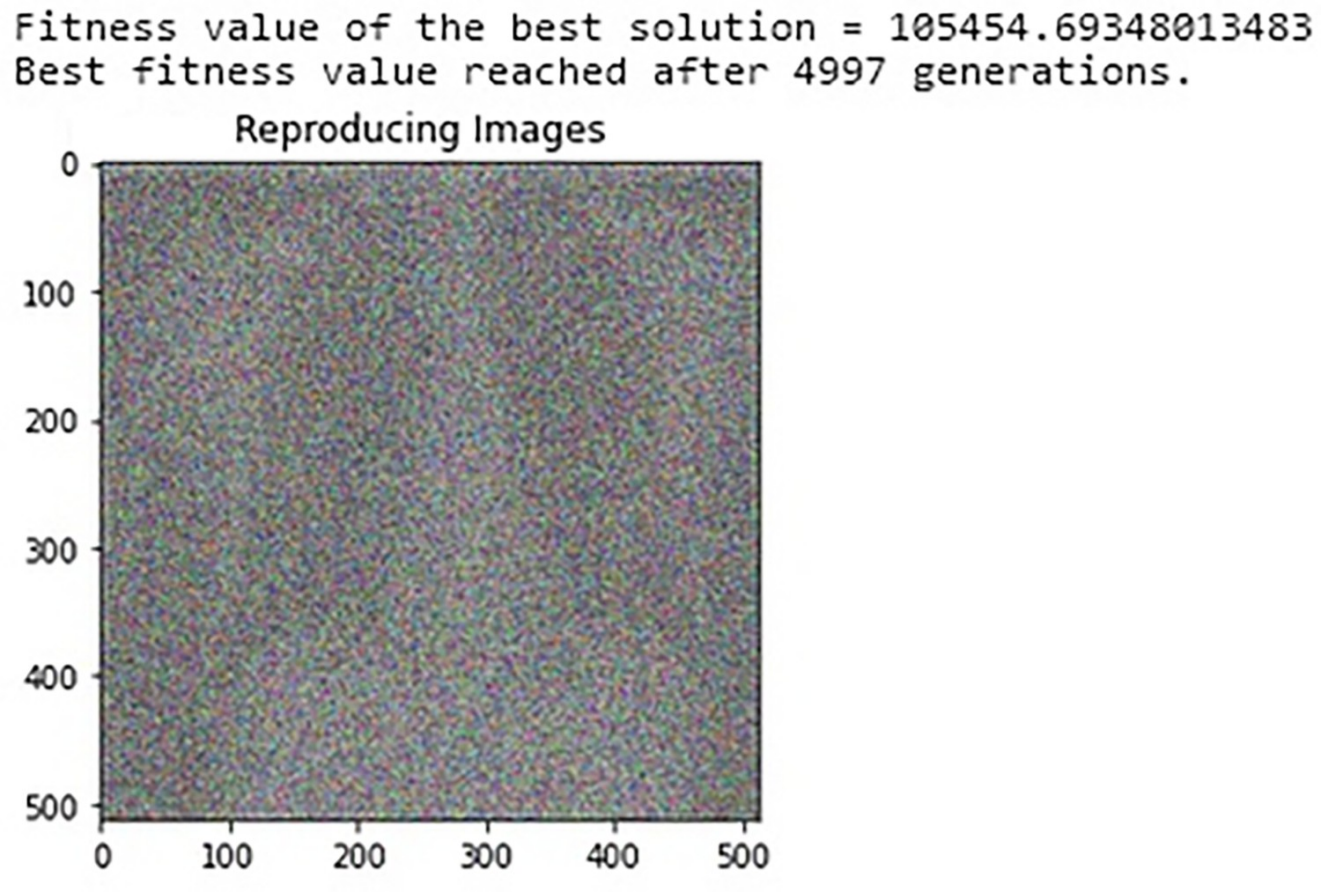
Image reconstruction.

The findings can even be improved by modifying the arguments given to such class’s function Object. Fig 5 below is showing a sample of source images which shows how it transformed after a few iterations.

Following that, Fine-tuned ResNet50 subsequently trained upon that basis all the preceding phases. The checkpoint has been set for the said model so that the best fitness results could be saved and the most recent best accuracy could be used. Finally, classification was performed by supplying the query image and then converting it with an array. An argmax function was used, that returns this index of the largest number within the given row and column, also with rows or columns selected based on both the argmax method’s axis property. This predict function describes the type of function provided that assists in generating output predictions using the specified sample of parameters onto a model.

**Fig 6:**
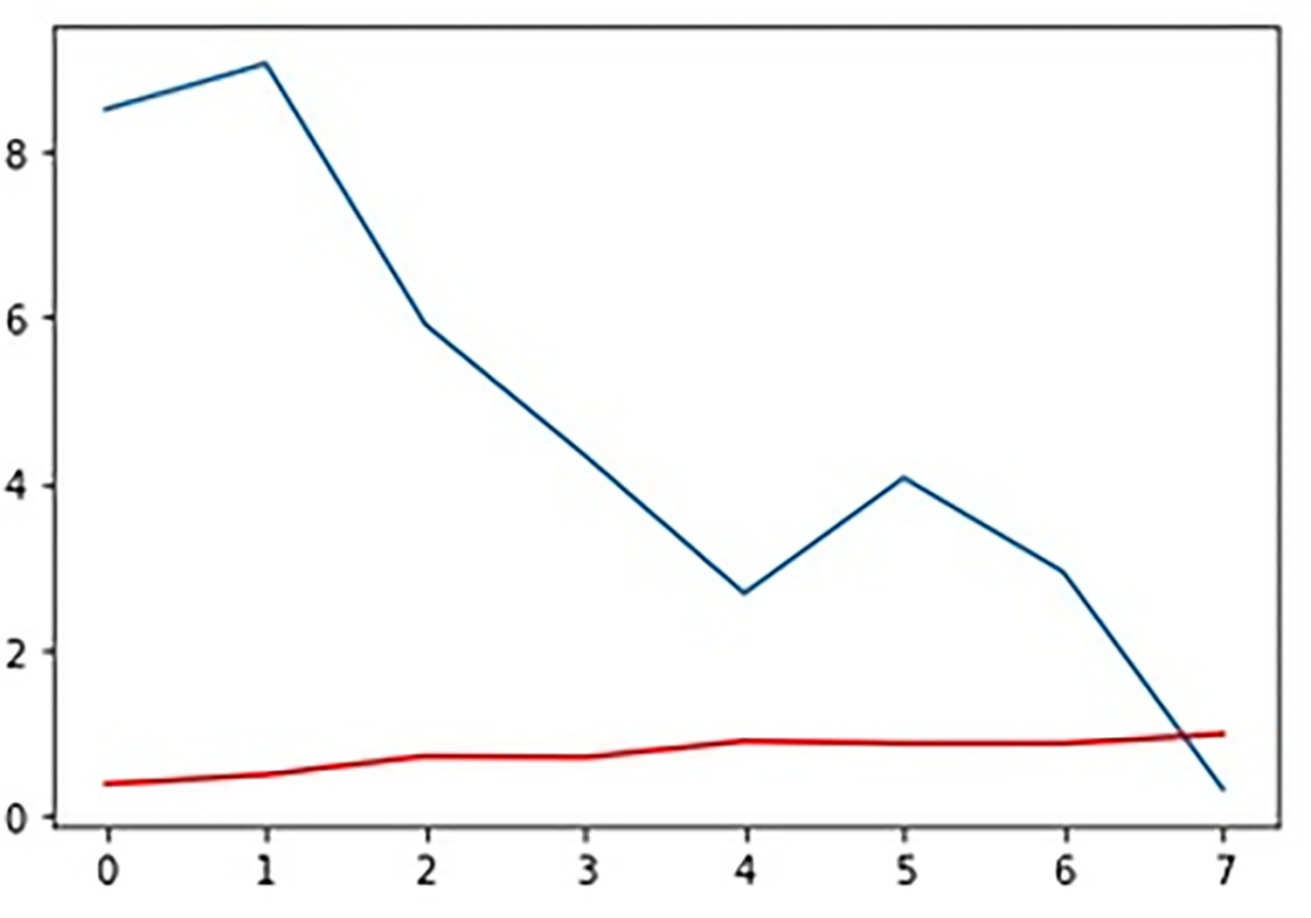
Accuracy vs loss function (Red = Accuracy, Blue = Loss function).

As a result of matching prediction with input arrays about image classification, the semantic gap significantly decreased. Overall training loss vs accuracy including both degrees for cross-validation for each epoch showed in Fig 6. After a certain epoch, the total loss has been 0.3304 across all configurations, but the prediction accuracy has hit 98.61%, suggesting that our ResNet50 CNN has also been properly trained to utilize training data. Moreover, after completing a set of CNN model training testing, we noticed that fine-tuning our model produces more accuracy versus standard training from the start.

## 5. Performance measure

To assess classification performance, metrics F1 score and precision matrix have been utilized. Evaluation metrics have been used to assess the classifier’s efficiency.

### 5.1 Accuracy metrics

The performance of the model including all classes is precisely measured. Overall accuracy is measured by adding the overall number of relevant guesses to the overall number of forecasts. Precision, recall, but also F1-Score have been calculated as performance parameters. The precision is stated as follows.


$$Accuracy=\frac{T_{p}+T_{n}}{T_{p}+T_{n}+F_{p}+F_{n}}$$ (9)

Where *TP* stands for True Positive, *TN* stands for True Negative, *FN* stands for False Negative, whereas *FP* is for False Positive. Our classifier performance is measured using evaluation measures.

Another essential statistic for assessing the algorithms is the F1 score. This is the fundamental accuracy and recall which is given as follows:

$$F1.score=2*\frac{Precision*Recall}{Precision+Recall}$$ (10)

**Fig 7:**
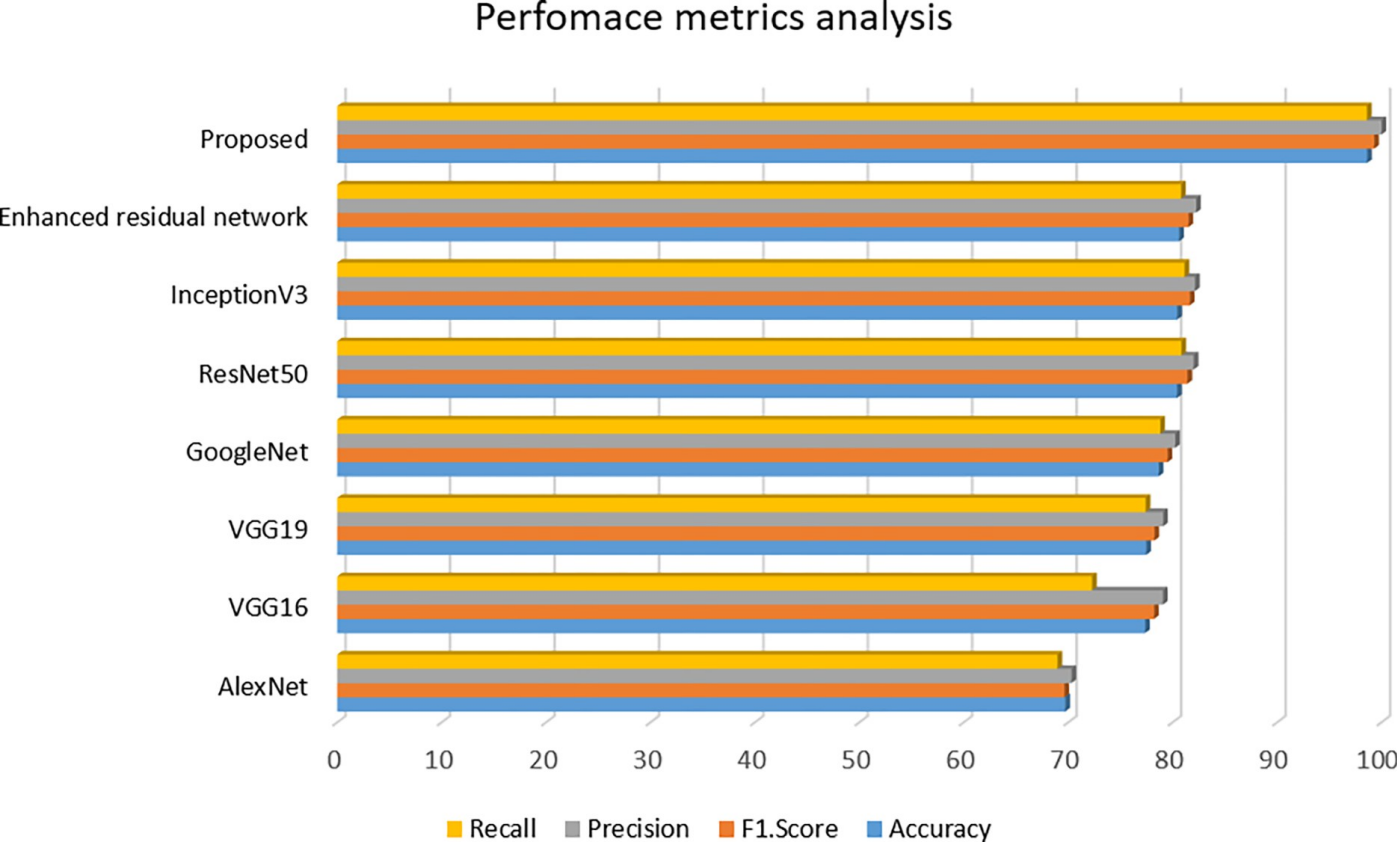
Performance metrics.

The influence about transfer learning has been examined through fine-tuning top 3 CNN models based on Table 3 outcomes using our improved deep residual modelling. The earlier ImageNet dataset had previously pre-trained those models [57]. This chosen collection fine-tuned the final few convolutions along with all of the FC layers over transfer learning, whereas the dataset provided by ImageNet optimized filter weights within the early convolutional layer training. In terms of precision, F1 score, accuracy, and Recall, our improved deep residual strategy outperformed the other models by using transfer learning. Table 3 and Fig 7 below show a comparison of various previous research and our model.

**Table 3:** Performance analysis of proposed and other models.

Model	Accuracy	F1.Score	Precision	Recall
AlexNet [58]	69.71	69.63	70.31	68.97
VGG16 [59]	77.36	78.22	79.06	72.27
VGG19 [59]	77.46	78.25	79.08	77.41
GoogleNet [60]	78.66	79.51	80.23	78.81
ResNet50 [61]	80.42	81.41	82.01	80.83
InceptionV3 [62]	80.43	81.63	82.13	81.14
Enhanced residual network [63]	80.62	81.50	82.24	80.79
**Proposed**	98.61	99.30	100	98.6

A t-test approach was used to investigate the relevance of our suggested model compared to the second-highest method, Enhanced residual network [63]. The Table 4 displays the t-test results for our suggested model with the second-highest method. The t-test study was predicated upon a test of null hypothesis, of which assumed that we have no significant difference regarding performance comparing our suggested model with the second-highest method. The results presented in Table 4 demonstrate that the significance levels of accuracy as well as F1. score during this test remained 0.0269 (below 0.05) as well as 0.02189 (below 0.05), respectively, while running a t-test. These findings indicating the null hypothesis regarding accuracy was not accepted at a 95% confidence level, indicating that there was indeed a significant disparity for accuracy comparing our model with the second-highest model. Furthermore, the null hypothesis regarding F1. score was not accepted given 95% confidence, demonstrating the significant improvement of our proposed model over the second-highest model.

**Table 4:** T-test for proposed and 2nd-best model (Enhanced residual network [63]).

Models	P value for Accuracy	P value for F1.Score
Enhanced residual network	0.0269 < 0.05	0.02189 < 0.05
Proposed		

Data pre-processing approaches including random rotation flips, and scale transformation, along with associated pre-processing operations, are utilized to expand the training set to ensure the variety of sample images and prevent over-fitting. These procedures are detailed further down.

1. Image resizing: For said model fitting, whole images were resized to 512 by 512-pixel resolution.
2. Image per-processing: used to reduce the various sequences of image data to bring these into ratios despite keeping the original image’s knowledge construction and striving to minimize image distortion.
3. Dataset separation and training. That section contains a collection of randomly sampled images for suggested tests and computed results.
4. Testing and validation. The images are used to assess the model being tested, and additional images from certain modelling are utilized to validate the model’s efficacy.

## 6. Comparison with chest X-ray dataset

**Fig 8:**
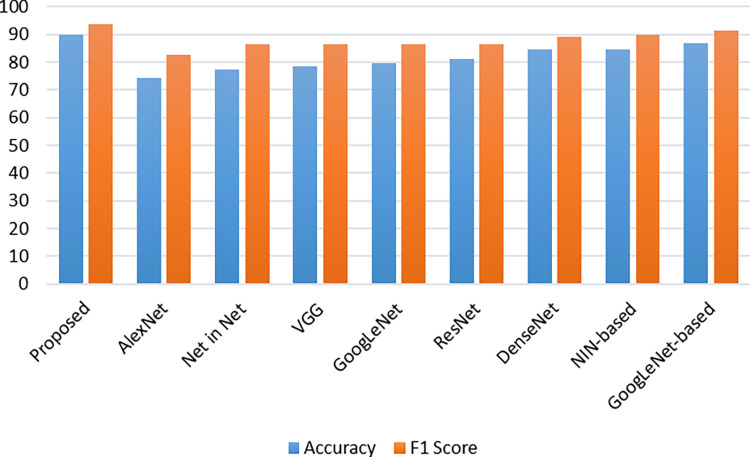
Performance comparison of multi-classification with chest X-ray dataset.

We tested our proposed model with existed model and dataset [64] in terms of creating a more direct comparison across our technique and cutting-edge approaches in real medical applications. Through this experiment, we executed multiclass classification on just a dataset containing Chest X-rays to replicate the treatment of our technique for identifying juvenile pneumonia. There were 5232 images in the dataset including normal and pneumonia. The Table below displays the results of the multiclass exercise performed on the dataset. Table 5 and Fig 8 showed that our technique outperforms existing approaches in general.

**Table 5:** Chest X-ray dataset performance comparison with various existing models.

Model	Accuracy	F1 Score
Proposed	90.0	93.67
AlexNet [65]	74.20 ±1.48	82.57 ±1.4
Net in Net [66]	77.40 ± 0.91	86.57±0.95
VGG [59]	78.53±0.96	86.68±1.01
GoogLeNet [67]	79.65± 0.91	86.34±1.47
ResNet [68]	81.25± 0.98	86.40±1.05
DenseNet [69]	84.53±0.83	89.02±0.94
NIN-based [64]	84.46±0.69	89.86±0.70
GoogLeNet-based [64]	86.70±0.82	91.26±0.87

## 7. Comparison with the Invasive Ductal Carcinoma (IDC) dataset

**Fig 9:**
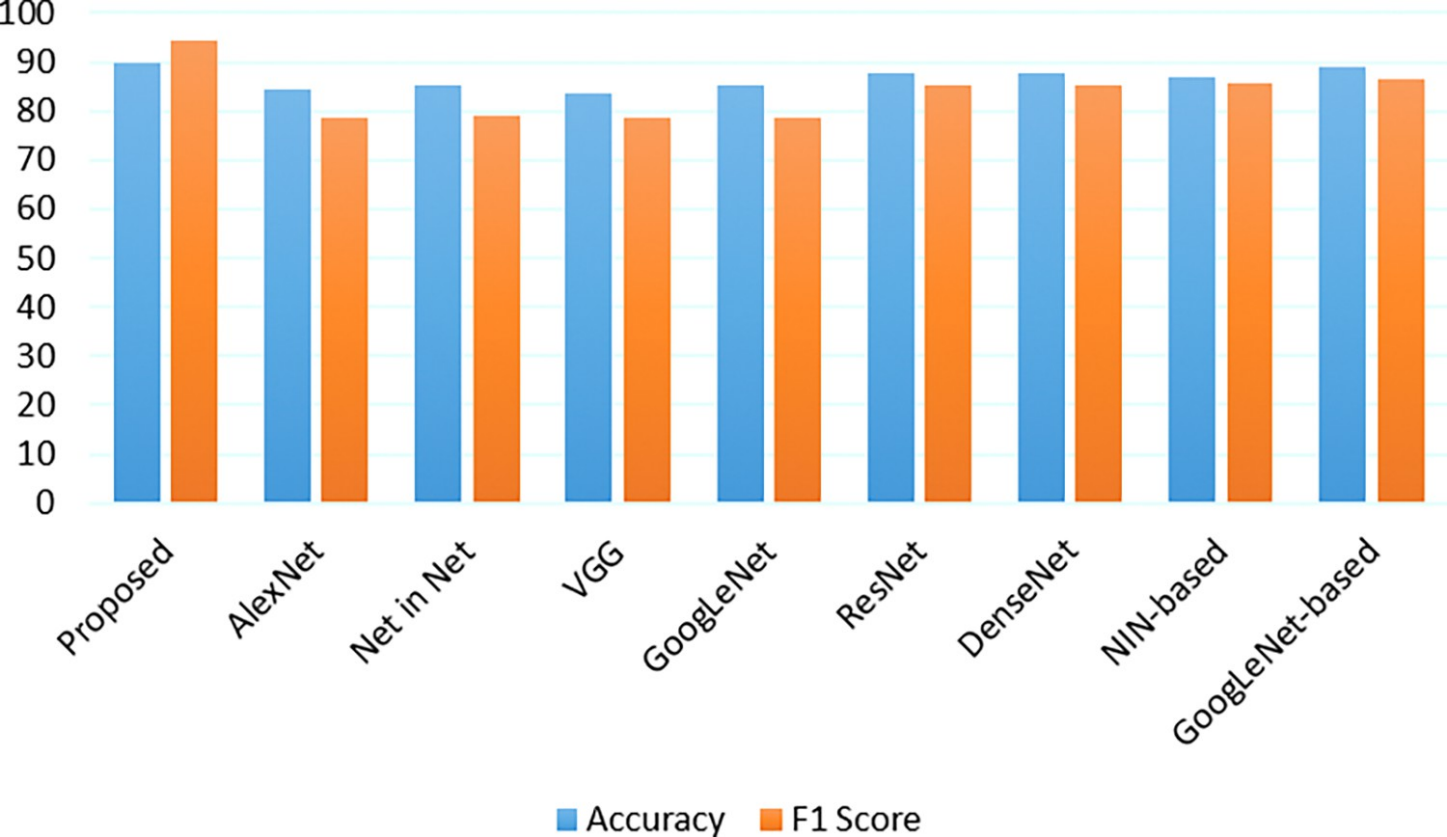
Performance comparison of multi-classification with IDC dataset.

IDC [70] represents the most prevalent form of breast cancer, accounting for over 80% of all cases. Unfortunately, because of the absence of distinguishing characteristics, it is challenging to identify IDC as just a distinct histological type such as lobular with tubular carcinoma. Throughout this study, we measure the effectiveness of our methods against the performances of CNNs mostly on the BHI dataset [71]. This collection contains 274 histopathology presentation images showing IDC tissue areas from 274 individuals, which were scanned using thought the entire scanner. Each experiment is run on subgroups of the complete BHI dataset using ready-to-use image patches. The very first collection includes 269 patient presentations with 272494 patched, comprising 76303 successful but also 196191 negatives. According to Table 6 and Fig 9, our strategy outperforms the other techniques in terms of efficiency and F1 measure. From several viewpoints, it has been demonstrated that CNNs achieve higher statistical parameters, indicating that their performances are un-stabilized because they are less sensitive to modeling initialization versus our techniques. Based on these findings, we may conclude that our model provided a viable technique for classification tasks using medical image datasets.

**Table 6:** IDC dataset performance comparison with various existing models.

Model	Accuracy	F1 Score
Proposed	90.0	94.49
AlexNet [65]	84.55 ±0.71	78.78 ±0.79
Net in Net [66]	85.16 ± 0.54	79.08±0.58
VGG [59]	83.77±0.69	78.42±0.65
GoogLeNet [67]	85.23± 0.49	78.74±0.52
ResNet [68]	87.54± 0.51	85.37±0.50
DenseNet [69]	87.62±0.45	85.22±0.56
NIN-based [64]	87.03±0.30	85.60±0.30
GoogLeNet-based [64]	88.88±0.25	86.33±0.22

## 8. Comparison with COVID19-CT dataset

**Fig 10:**
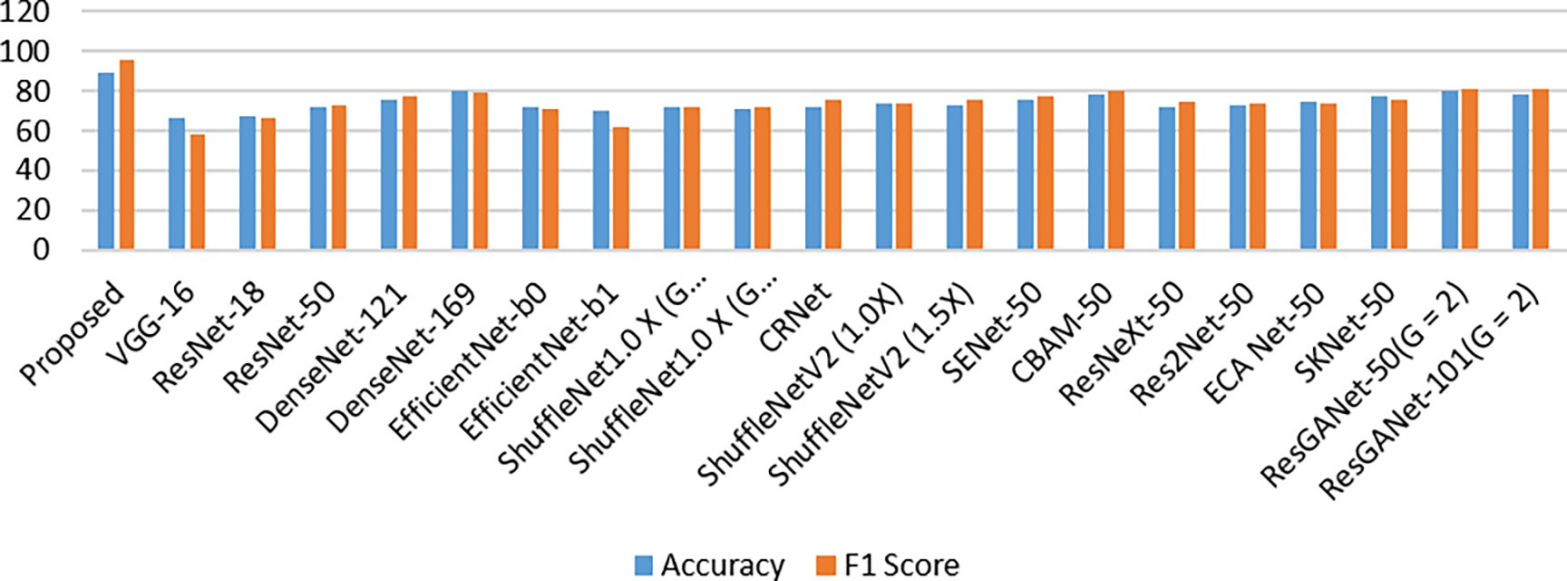
Performance comparison of multi-classification with COVID19-CT dataset.

This COVID-19-CT dataset [72] includes various medical images gathered by [73]. It contained 349 COVID-19 realistic CT scan images with 397 normal but rather negative CT scan images from different disorders. The images in this collection varied in size between 143 × 76 through 1637 × 1225. Our assessment findings from the proposed method and many of the most sophisticated classification methods upon that COVID-19-CT dataset are shown in Table 7. Our experimental results confirmed whether deeper or broader networks typically exhibited better classification performance, which was aided by the complicated network topology. The suggested model’s efficiency and F1 value achieved the greatest performance throughout this dataset, according to Fig 10 below.

**Table 7:** COVID19-CT dataset performance comparison with various existing models.

Model	Accuracy	F1 Score
Proposed	88.89	95.35
VGG-16 [59]	66	58
ResNet-18 [74]	67	66
ResNet-50 [74]	72	73
DenseNet-121 [69]	76	77
DenseNet-169 [69]	80	79
EfficientNet-b0 [75]	72	71
EfficientNet-b1 [75]	70	62
ShuffleNet1.0 X (G = 4) [76]	72	72
ShuffleNet1.0 X (G = 8) [76]	71	72
CRNet [73]	72	76
ShuffleNetV2 (1.0X) [76]	74	74
ShuffleNetV2 (1.5X) [76]	73	76
SENet-50 [77]	76	77
CBAM-50 [78]	78	80
ResNeXt-50 [79]	72	75
Res2Net-50 [80]	73	74
ECA Net-50 [81]	75	74
SKNet-50 [82]	77	76
ResGANet-50(G = 2) [83]	80	81
ResGANet-101(G = 2) [83]	78	81

## 9. Comparison with ISIC2018

**Fig 11:**
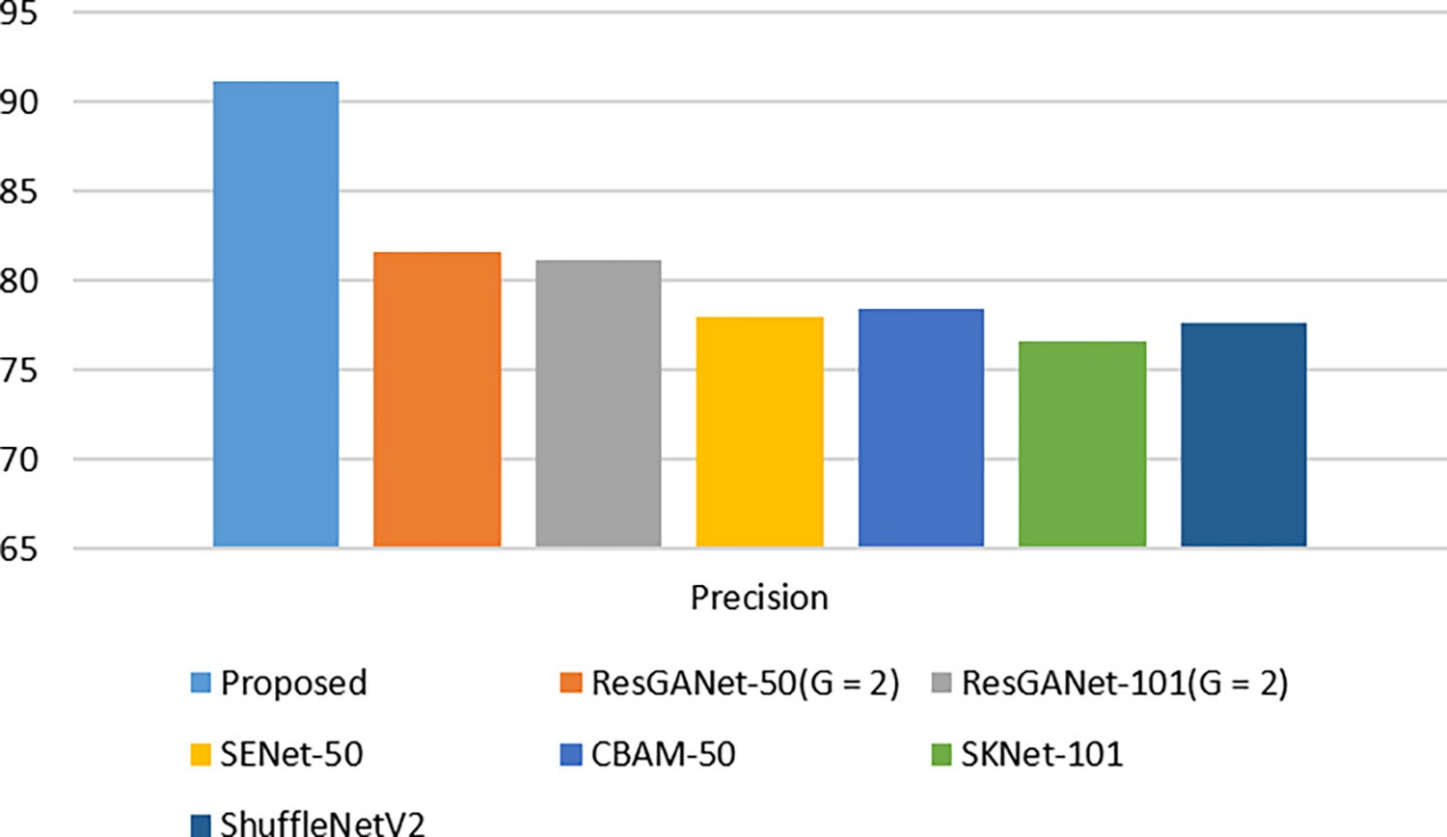
Performance comparison of multi-classification with ISIC2018 dataset.

The ISIC2018 [84] skin lesion diagnostic dataset has been used. This dataset has a total number of 10,015 images divided into seven subcategories. Melanocytic nevus (6705), melanoma (1113), dermatofibroma (115), benign keratosis (1099), actinic keratosis (327), vascular lesion, and basal cell carcinoma (514) become the names of these conditions (142). To make comparisons between ResNet and its derivatives [76, 77, 82, 85]. In each task, we explored 50- as well as 101-layer models. Furthermore, we compared several lightweight techniques. The difficulty level with Shuffle Net [86] is 1.0. These findings demonstrated in Table 8 and Fig 11 that the suggested model outperforms the most sophisticated ResNet variants network upon this ISIC2018 dataset for medical image categorization.

**Table 8:** ISIC2018 dataset performance comparison with various existing models.

Model	Precision
**Proposed**	**98.82**
ResGANet-50(G = 2) [83]	81.66
ResGANet-101(G = 2) [83]	81.13
SENet-50 [77]	77.97
CBAM-50 [85]	78.47
SKNet-101 [82]	76.61
ShuffleNetV2 [76]	77.7

## 10. Conclusion and future work

In this research, the multi-modal medical image collection was generated using available public images. The convolutional neural network-based ResNet50 framework is subjected to data enhancement, database pre-processing, training, then testing approaches. The suggested model was developed and tested to enhance the performance that is assessed and compared.

When compared to most accessible datasets and approaches, the evaluation measurement parameters are relatively high and enhanced. As a result, our recommended research study significantly improved by 98.61%. Regularly enhancing the quality of multi-modal medical image evaluation and classification has become an essential part, but this model attained the maximum performance, assisting in the success of certain health sectors. The primary goal of the study is to enhance the health service. The future goal is to acquire and prepare actual datasets to be utilized in deep learning models including adversarial attacks. It is expected that various CNN models will be applied in the future with deeper image evaluation. Our work fosters and stimulates the health industry, which leads to an increase in medical education.

## 11. Limitations

The current research work is related to classification of multi modal medical images. Our dataset contained five types of medical images (i.e. endoscopy, CT, chest, hand x-ray, and lungs CT). Our model is only optimally trained for said five types of images and its accuracy can be affected if different image class included in dataset. Furthermore, our model is only optimally trained, and it is not a robust model that counters adversarial image attacks.

## Supporting information

S1 File (RAR)
